# Validation of Catquest-9SF Questionnaire in a Chinese Cataract Population

**DOI:** 10.1371/journal.pone.0103860

**Published:** 2014-08-01

**Authors:** Xianchai Lin, Mingge Li, Mei Wang, Yajing Zuo, Siping Zhu, Yongxin Zheng, Xiaofeng Lin, Minbin Yu, Ecosse L. Lamoureux

**Affiliations:** 1 State Key Laboratory of Ophthalmology, Zhongshan Ophthalmic Center, Sun Yat-sen University, Guangzhou, China; 2 Department of Ophthalmology, Sun Yat-sen Memorial Hospital, Sun Yat-sen University, Guangzhou, China; 3 Shantou Central Hospital, Shantou, China; 4 Singapore Eye Research Institute, Singapore National Eye Centre, Singapore, Singapore; 5 Duke-National University of Singapore Graduate Medical School, Singapore, Singapore; 6 Centre for Eye Research Australia, University of Melbourne, Melbourne, Australia; Medical College of Soochow University, China

## Abstract

**Purpose:**

To develop and validate a Chinese version of the Catquest-9SF questionnaire in a cataract population.

**Methods:**

The Catquest-9SF Questionnaire was translated and back translated into Chinese. Preoperative patients were recruited at a tertiary eye hospital and their demographic information and visual acuity were documented. Psychometric properties of the Catquest-9SF, including ordered thresholds, the ability to distinguish between different strata of person ability, absence of misfitting items, unidimentionality, differential item functioning (DIF) and construct validity were tested, using Rasch analysis.

**Results:**

A total of 102 patients (100% response rate) were enrolled. The participants'mean age was 70.2 year (SD = 12.1) and 46.9% were female. Rasch analysis showed that this version of the questionnaire had ordered response thresholds and was free of DIF. The items fit a single overall construct and unidimensional by principal components analysis of the residuals. Patients with visual impairment had significantly poorer Rasch scores on the Catquest-9SF (mean change, -2.5, p = 0.035, compared with non-visually impaired patients).

**Conclusion:**

The Chinese version of Catquest-9SF is a valid and reliable questionnaire for assessing the visual disability outcomes of Chinese patients with cataract, and it may be recommended for routine clinical use.

## Introduction

Cataract is the leading cause of visual impairment and blindness; and is one of the most costly eye problems in today's economies. [1] Cataract surgery is a highly cost-effective medical procedure that results in improvement of visual acuity and vision-related quality of life. [2] While most patients can restore their best possible distant visual acuity through phacoemulsification cataract surgery and intraocular lens implantation, the recent advancements in lens designs (e.g., multifocal lens) and surgical technologies (e.g., femtosecond laser-assisted cataract surgery) have the potential to achieve better refractive outcome and more predictable quality of vision. [3] These new technologies highlight a need to identify indicators of both preoperative and postoperative visual function in order for ophthalmologists to determine whether a new intervention is worth the cost. [4] Additionally, accurate assessment of visual functioning is important for cataract patients to make an informed surgical decision and have a realistic expectation about the treatment [5].

A large number of vision-related functional questionnaires (e.g., NEI-visual functioning questionnaire 25 (NEI-VFQ 25), [6]; [7] visual function 14 (VF-14), [8]–[10] and activities of daily vision scale for cataract surgery (ADVS)) [11]–[13] have been previously developed, but these questionnaires showed suboptimal targeting and may need additional items to facilitate measurement. More recently, the Catquest questionnaire was developed to assess patients' perceived difficulties in daily life as a result of cataract. [14]–[17] The original Catquest questionnaire contains 12 items and is based on the presence of indicators in four areas: frequency of performing activities (7 items), driving (1 item), questions about difficulties in general and satisfaction with vision (2 items) and cataract symptoms (2 items). However, previous studies showed that the Catquest questionnaire was not unidimensional and the symptoms and frequency of performing activities items did not form valid subscales. [14]–[17] Therefore, its short form, the Catquest-9SF questionnaire, was developed to address these issues. [18], [19] Modern psychometric analyses confirmed the validity and reliability of Catquest-9SF questionnaire and showed that it was highly responsive to surgical treatment and the score was moderately associated with visual acuity [18], [19].

The Catquest-9SF questionnaire was originally developed in Swedish and the translation has only been cross-culturally validated in an English-speaking population in Australia. [17] China is home to the world's largest number of cataract patients, [20] but a Chinese version of the Catquest-9SF questionnaire has not yet been validated. To assess patients' perceived visual functioning in a new country, the questionnaire must be translated and validated using rigorous psychometric methods. The goal of this study was to translate and validate the Chinese version of the Catquest-9SF questionnaire in a Chinese cataract population.

## Methods

### Study population

One hundred and two patients awaiting cataract operation were recruited from Zhongshan Ophthalmic Center, Sun Yat-sen University, Guangzhou, China between June 2013 and November 2013. The patients underwent a comprehensive ophthalmic examination that included best-corrected visual acuity, biomicroscopy, intraocular pressure, and funduscopy. All patients completed the Catquest-9SF questionnaire and an additional questionnaire for information about patients' past medical history, demographic characteristics, health literacy, and educational level. The study was approved by the Institutional Review Board of Zhongshan Ophthalmic Center and adhered to the tenets of the Declaration of Helsinki. The survey was explained in details that it was voluntary and would not influence care, and thus informed consent was not obtained.

### Catquest-9SF questionnaire

The Catquest-9SF questionnaire consists of 7 questions for performing daily-life activities and 2 global questions about difficulties in general and satisfaction with vision. For each question, the response options are as follows: 1 =  very great difficulty; 2 =  great difficulty; 3 =  some difficulty; 4 =  no difficulty. Lower scores generally indicate better visual functions. The instruction information and the response formats presented in the current study represent a Chinese translation.

### Development of Catquest-9SF questionnaire

The development of Catquest-9SF questionnaire involved the following steps:

1. Two translators independently carried out the translation of the questionnaire (from English to Chinese). They also made specific comments to highlight challenging phrases and uncertainties.
2. Reconciliation of the Chinese translations was made by a senior ophthalmologist to provide the first draft of Catquest-9SF questionnaire.
3. A third translator (who had no knowledge of the original English version of the Catquest-9SF) translated the drafted Catquest-9SF questionnaire into English. The back-translated Catquest-9SF was compared with the original English version to identify discrepancies so that revision can be made to make sure that the translation reflected the same item content as the original one. This step resulted in a revised version of the Chinese translation of the Catquest-9SF questionnaire.
4. The Chinese version of the questionnaire was tested on two ophthalmologists and 4 persons with cataract randomly selected from the outpatient clinics to collect information about any difficulties in completing the questionnaire and to determine if the purpose or meaning of each question can be accurately understood. After minor revision, the final version of the questionnaire was established (available upon request).

### Rasch analysis

Rasch analysis is a psychometric method that places both item difficulty (item measure; i.e., how difficult the item is) and person ability (person measure; i.e., the extent to which person possesses the trait being tested) on the same scale, measured in log of the odds (logits) units. [21] In Rasch modeling, the probability of correctly answering a particular item is modeled as a logistic function of the difference between the person and item parameter. The model can be mathematically described as follows: Probability  = 1/(1+Exp(-(ability-difficulty))). When the person's ability “matches” the item difficulty, he or she has a 50% chance to answer the item correctly. [22] Rasch analysis gives information on both persons and items, and it provides insight into the psychometric properties of the scale. It determines how well items (a) fit the latent trait (e.g., vision function) being measured, (b) discriminate between respondents, and (c) target person ability. [23] In the current study, Rasch analysis was used to validate the Chinese version of the Catquest-9SF questionnaire based on the Andrich rating scale model (joint maximum likelihood estimation) using WINSTEPS software (version 3.63.2, Chicago, IL). [24] Several indicators were used to assess the validity of the questionnaire. These included: (1) person separation index (PSI). PSI provides an estimate of the discriminating ability of the items between strata of person ability. A PSI of 2.0 and a person reliability (PR) score of 0.8 may be considered sufficient for discrimination of at least 3 strata of persons' level of the trait being measured. (2) Person-item map. The map shows person measures ranked by their ability level and item difficulties ranked by difficulty. It provides a way to visualize how well the items target the ability of the sample. Ideally, the mean person measure should be approximately 0 logits. A difference between the mean person and item measure of more than 1.0 logits generally indicates significant mistargeting. (3) “infit” mean square (MNSQ). This is used to indicate the ability of a scale to measure a single latent trait. A MNSQ value between 0.7 and 1.3 were considered acceptable for unidimensionality. Items outside this range can be removed from the scale to improve fit. (4) Principal component analysis (PCA) of the residuals. PCA is used in combination with Rasch fit statistics to test the unidimensionality of the measured trait. The PCA transforms correlated items into principal components whereby the variance explained by the measures for the empiric calculation should be comparable with that of the model (>50%). An unexplained variance in the first contrast of the residuals <3.0 eigenvalue units suggests the nonexistence of a secondary trait captured by the instrument. (5) Differential item functioning (DIF). This measure is used to indicate if different groups (stratified by age, sex, or educational level) have systematically different responses despite having equal levels of trait being measured. A DIF value >1.0 logits is considered significant, and thus the interpretation of the results should be stratified by groups [25]–[28].

## Results

### Sociodemographic and clinical characteristics of the study population

A total of 102 people (100% response rate) participated in the study (**Table 1**). Overall, the participants' mean age was 70.2 year (SD = 12.1) and 46.9% were female. Using best-corrected data in the better-seeing eye, 70 (68.6%) of the patients had visual impairment.

**Table 1:** Characteristics of the participants.

Characteristics	N (%)
Participants	102
Sex	
Male	52 (53.1)
Female	46 (46.9)
Age	
< = 60	19 (18.6)
>60	83 (81.4)
Education	
No formal education or primary school	41 (40.2)
Secondary or high school	51 (50.0)
University or higher	10 (9.8)
Income per month	
<RMB$3000	54 (52.9)
≥RMB$3000	48 (47.1)
Diabetes (yes)	6 (6.1)
Hypertension (yes)	40 (40.4)
Best-corrected visual impairment	
No	70 (68.6)
Yes	32 (31.4)
Self-reported health rating	
Poor to fair	17 (16.7)
Good	43 (42.2)
Very good to excellent	42 (41.2)

### Threshold order and person separation

**Figure 1:**
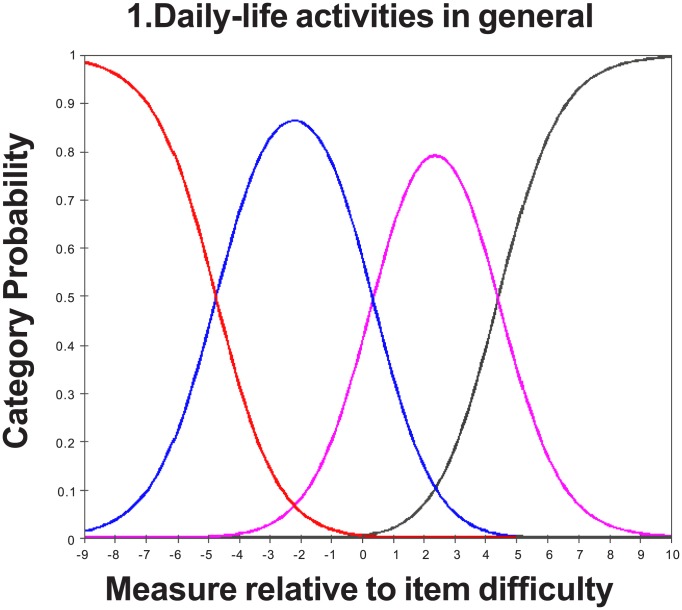
Category probability curves for the global “difficulties in your daily life” item, which illustrates ordered threshold.

The category probability curves showed that there was no evidence of disordered thresholds (**Figure 1**). The PSI and PR values were 2.94 and 0.90, respectively, suggesting good discriminant ability of the questionnaire.

### Unidimensionality

The ‘infit’ MNSQ values for the items were between 0.7 and 1.3 (**Table 2**), showing acceptable fit to the model expectation. The PCA of the residuals explained 63.0% of the raw variance, suggesting that there was no evidence of multidimensionality in the scale. The unexplained variance in 1st contrast was 1.9 eigenvalue units, and thus there was no evidence of another latent trait captured by the scale.

**Table 2:** Item infit statistics and item difficulty rating (logits).

Items	Infit mean square	
	Australia	China
Daily-life activities in general	0.86	1.22
Satisfaction with vision in general	1.19	1.05
Read newspaper	0.76	1.18
Recognize faces	1.22	0.84
Prices when shopping	0.86	0.82
Walk on uneven ground	0.98	0.97
Needlework	0.91	0.96
Seeing text on TV	1.21	1.03
Hobbies	1.01	0.82

### Person-item map

**Figure 2:**
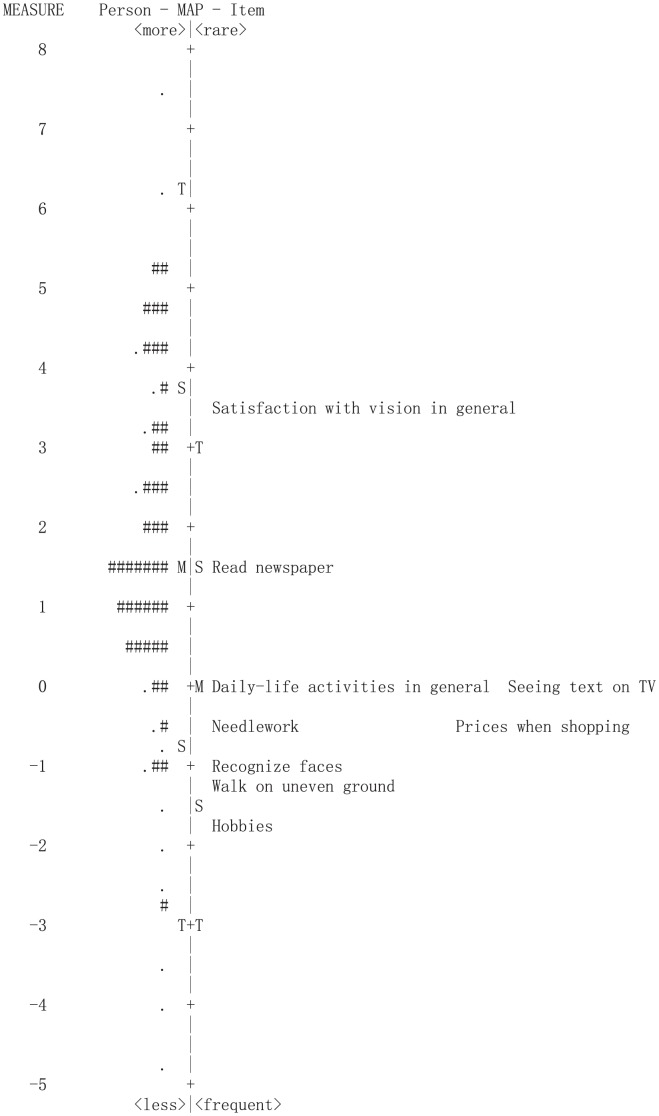
Person-item map of the Chinese version of the Catquest-9SF.

Both the person and item scores were rescaled and are shown on the person-item map (**Figure 2**). Persons with the highest level of ability are located at the top of the diagram while those with the lowest level are at the bottom. On the right side of the dash line are the items, and items that are easier to perform further down the scale. There was an adequate spread of items across the range of person ability, although most of the items were located at the low end of the scale. Also, the difference in item and person mean was 1.61 logits. These results suggested that the items were generally too easy to perform for this population.

### Differential item functioning (DIF)

One of the requirements of Rasch model is that the scale should function consistently across different subgroups (e.g., age, sex and education level). In other words, the subgroups should respond in the same way to the items. Notable DIF was seen for item 8 - difficulty seeing text on TV (i.e., people aged 60 years and over were generally more able to carry out this task than those younger than 60 years by 1.25 logits), and item 3 - difficulty reading newspaper (i.e., people with an university-level education were more able to read newspaper than those with no formal or primary education). Nevertheless, the majority of items functioned similarly for participants at the same level of ability (**Table 3**).

**Table 3:** Differential item functioning (DIF) by age, sex, and education level.

	DIF by age				DIF by sex				DIF by education			
	< = 60 y	>60 y	Contrast	Welch's test	Male	Female	Contrast	Welch's test	No or primary education	University	Contrast	Welch's test
Daily-life activities in general	0.78	−0.22	1.00	0.0656	0.20	−0.34	0.54	0.2073	−0.15	0.79	−0.94	0.2090
Satisfaction with vision in general	4.58	3.26	1.32	0.0310	3.59	3.37	0.22	0.6310	2.55	4.06	−1.51	0.0685
Read newspaper	0.78	1.71	−0.93	0.0906	1.71	1.42	0.30	0.4892	1.99	−0.03	2.02	0.0118
Recognize faces	−1.15	−0.90	−0.25	0.6605	−1.09	−0.64	−0.44	0.3061	−1.00	−0.85	−0.15	0.8404
Prices when shopping	−0.63	−0.46	−0.17	0.7605	−0.30	−0.70	0.40	0.3586	−0.38	−0.68	0.30	0.6970
Walk on uneven ground	−1.15	−1.40	0.25	0.6679	−1.35	−1.49	0.14	0.7533	−1.32	−0.44	−0.89	0.2315
Needlework	−0.14	−0.67	0.52	0.3408	−0.60	−0.50	−0.10	0.8157	−0.26	−1.16	0.90	0.2601
Seeing text on TV	−1.04	0.20	−1.25	0.0378	−0.47	0.46	−0.93	0.0296	0.49	−0.03	0.53	0.4703
Hobbies	−2.06	−1.57	−0.50	0.4185	−1.70	−1.62	−0.08	0.8619	−2.10	−1.74	−0.36	0.6439

### Criterion validity and reliability

We assessed the questionnaire's criterion validity by examining its ability to discriminate subgroups with different levels of visual acuity. Persons with worse visual functioning score were significantly associated with presence of visual impairment (mean change, −2.5, p = 0.035, compared with non-visually impaired patients). Cronhach α of the items ranged from 0.86–0.88 (**Table 4**).

**Table 4:** Internal consistency of the Catquest-9SF items.

Items	Cronbach α
Daily-life activities in general	0.88
Satisfaction with vision in general	0.88
Read newspaper	0.86
Recognize faces	0.86
Prices when shopping	0.86
Walk on uneven ground	0.87
Needlework	0.87
Seeing text on TV	0.86
Hobbies	0.87

## Discussion

Our results show that the Chinese Catquest-9SF is a unidimensional, reliable and valid questionnaire that is useful for assessing visual functioning in Chinese patients with cataract. Our Rasch model confirmed the findings from previous studies in Sweden and Australia that the Catquest-9SF scale has ordered thresholds and slight mistargeting, and was free of any large DIF. Nevertheless, there is a need to advocate the application of this questionnaire, which is not commonly used in clinical practice, presumably due to the fact that many clinicians still consider it as a research-tool, rather than a means to identify patients with impaired visual function.

One of the advantages of the Catquest-9SF questionnaire is that it is not lengthy compared with other visual functioning questionnaires (e.g., ADVS and VF-14). [8]–[13] With 9 items, it is relatively easy and time effective to administer and receive responses from the participants. By contrast, the original Catquest questionnaire has 12 items that include disability (7 items), global assessments (2 items), symptoms (2 items), and driving (1 item). The original Catquest questionnaire was found to be multidimensional for the Swedish cataract patients. [14]–[17] The removal of the symptom and driving items improved fit, and thus leading to the development of Catquest-9SF questionnaire [18], [19].

The person-item map shows the distribution of participants' ability and item difficulty attributes. Although there was a fairly even spread of items about the ability continuum, the Catquest-9SF items showed slight mistargeting, with some participants possessing greater abilities than required by the most difficult item. These results suggested that the Catquest-9SF items were too easy for the visual abilities of the participants. Further studies are needed to assess if the inclusion of additional items can result in better targeting of items for the visual abilities of the participants. Similar to findings in the Australian cohort, item 2 (“satisfaction with one's present vision”) was found to be the most difficult question, whereas item 9 (“hobbies”) was the easiest one [17].

There was no large DIF for the majority of the items in the current study. Nevertheless, item 8 (“seeing text on TV”) showed significant DIF across different age and gender groups. One possible explanation is that the frequency of performing this activity may be different among different subgroups. It is possible that this activity is more commonly performed among older people and among females in China, and thus older people and women may be more likely to experience the difficulty than young persons and men. In addition, item 3 (“reading newspaper”) showed significant DIF was for subgroups with different education levels. This is explicable on the ground that people with no formal or with primary education are far less likely to read newspaper than those with a University diploma. These items were retained in the questionnaire, given that the DIF was not substantial.

The strengths of this study include the use of Chinese patients with cataract with a useful age distribution, reasonable representation of both sexes, and the use of modern psychometric theory to validate the scale. Nevertheless, this study has a few limitations. First, we did not assess the responsiveness of the questionnaires (e.g., change in visual functions after cataract surgery). Second, all patients were enrolled from a single hospital. They may not be fully representative of the general population in China. All were hospitalized and waiting for cataract surgery, and may suggest that that the majority of them (87%) were not satisfied with their present vision. Third, while we believe that this version of the Catquest-9SF questionnaire could be used without further adaptation in most of the Chinese-speaking regions (e.g. Singapore, Taiwan, etc.), a cultural adaptation of the questionnaire may still be needed outside of China.

In conclusion, we found that the Chinese version of the Catquest-9SF questionnaire is valid and reliable, and is linguistically and culturally suitable for use among Chinese speaking patients in China. The Chinese version of the Catquest-9SF is easy to understand and quick to complete, and will serve as an important tool to assess vision function for patients with cataract in Chinese-speaking communities.
